# Preparation and Characterization of Undecylenoyl Phenylalanine Loaded-Nanostructure Lipid Carriers (NLCs) as a New α-MSH Antagonist and Antityrosinase Agent

**DOI:** 10.34172/apb.2023.036

**Published:** 2022-01-08

**Authors:** Mohadeseh Sadat Vaziri, Zahra Tayarani-Najaran, Homa Kabiri, Samira Nasirizadeh, Shiva Golmohammadzadeh, Hossein Kamali

**Affiliations:** ^1^Student Research Committee, School of Pharmacy, Mashhad University of Medical Sciences, Mashhad, Iran.; ^2^Targeted Drug Delivery Research Center, Pharmaceutical Technology Institute, Mashhad University of Medical Sciences, Mashhad, Iran.; ^3^Medical Toxicology Research Center, Mashhad University of Medical Sciences, Mashhad, Iran.; ^4^School of Pharmacy, Mashhad University of Medical Sciences, Mashhad, Iran.; ^5^Nanotechnology Research Center, Pharmaceutical Technology Institute, Mashhad University of Medical Sciences, Mashhad, Iran.; ^6^Department of Pharmaceutics, School of Pharmacy, Mashhad University of Medical Sciences, Mashhad, Iran.

**Keywords:** Sepiwhite, Undecylenoyl phenylalanine, Melanogenesis, Nanostructured lipid carriers, Permeation study, Brightener

## Abstract

***Purpose:*** The aim of this study was to characterize the undecylenoyl phenylalanine (Sepiwhite (SEPI))-loaded nanostructured lipid carriers (NLCs) as a new antimelanogenesis compound.

***Methods:*** In this study, an optimized SEPI-NLC formulation was prepared and characterized for particle size, zeta potential, stability, and encapsulation efficiency. Then, *in vitro* drug loading capacity and the release profile of SEPI, and its cytotoxicity were investigated. The *ex vivo* skin permeation and the anti-tyrosinase activity of SEPI-NLCs were also evaluated.

***Results:*** The optimized SEPI-NLC formulation showed the size of 180.1±5.01 nm, a spherical morphology under TEM, entrapment efficiency of 90.81±3.75%, and stability for 9 months at room temperature. The differential scanning calorimetry (DSC) analysis exhibited an amorphous state of SEPI in NLCs. In addition, the release study demonstrated that SEPI-NLCs had a biphasic release outline with an initial burst release compared to SEPI-EMULSION. About 65% of SEPI was released from SEPI-NLC within 72 h, while in SEPI-EMULSION, this value was 23%. The *ex vivo* permeation profiles revealed that the higher SEPI accumulation in the skin following application of SEPI-NLC (up to 88.8%) compared to SEPI-EMULSION (65%) and SEPI-ETHANOL (74.8%) formulations (*P*<0.01). An inhibition rate of 72% and 65% was obtained for mushroom and cellular tyrosinase activity of SEPI, respectively. Moreover, results of *in vitro* cytotoxicity assay confirmed SEPI-NLCs to be non-toxic and safe for topical use.

***Conclusion:*** The results of this study demonstrate that NLC can efficiently deliver SEPI into the skin, which has a promise for topical treatment of hyperpigmentation.

## Introduction

**Figure 1:**
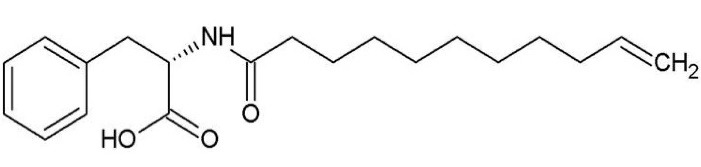
Chemical structure of undecylenoyl phenylalanine (Sepiwhite^®^).

 Stratum corneum (SC) is the first layer of epidermis, which is comprised of dehydrated and keratin-rich corneocytes compacting in a continuous lipid bilayer. This layer is the main barrier against the penetration of water-soluble and lipid-soluble materials into the skin.^1^ The stratum basale is the innermost layer of the epidermis that contains melanocytes, melanin-producing cells.^2^ Melanin is a skin color determining and ultraviolet radiation (UV) protection factor.^3,4^ In melanocytes, melanin pigments are synthesized from tyrosine through the enzymatic process called melanogenesis.^5^ This process is multifactorial that is regulated by various factors, consisting of the female sex hormones, melanotropin or α-melanocyte-stimulating hormone (α-MSH), and catecholamines. Hormone α-MSH and catecholamines induce melanogenesis through type 1 melanocortin receptors (MC1Rs) and β-adrenergic receptors (β-ADRs), respectively. These hormones are secreted in response to sunlight, UV, hormonal influences, and other environmental stimulating factors. The overproduction or abnormal distribution of melanin can cause freckles, melasma, and hyperpigmentation.^3,4^ Therefore, the inhibition of α-MSH and β-ADR can be a potent strategy for treating hyperpigmentation. Undecylenoyl phenylalanine (Sepiwhite^®^, abbreviated to SEPI)) (Figure 1) is a novel lightening agent, which developed inspired from natural MC1R receptors antagonists presenting in the skin, AGRPs (agouti-related protein) and probably acting as an antagonist of α-MSH and β-ADR.^5,6^ In recent years, SEPI has been used to treat melisma.^7-9^

 The treatment of local cutaneous dermatologic by employing a pharmaceutical agent is easy, suitable, and generally well accepted by the patients.^10^ However, the prevention of dermal drug penetration by the SC layer is the main limitation of this strategy. There is also convincing evidence indicating that the drug released from the conventional formulations is usually trapped in the upper layers of SC and cannot pass through it.^11^

 Among modern drug delivery carriers, nanostructured lipid carriers (NLCs) are promising colloidal carriers. Some advantages of NLCs are the protection of the encapsulated agents against chemical and enzymatic degradation, good stability during the storage period, avoidance against organic and alcoholic solvent, easy and scalable production process, controlled release profile of drugs, and enhancing the penetration of drugs into the skin.^12^ Controlled drug release is vital for long-time drug delivery and regulates the systemic absorption of the drug, which is important when the drug is stimulant in high concentrations.^13^ Moreover, NLCs have a higher encapsulation capacity, a lesser drug leakage, and an improved drug release profile compared to solid lipid nanoparticles (SLNs) due to the partial replacement of solid lipid with liquid lipid. It has been demonstrated that lipid nanoparticles (NPs) have UV-blocking effects.^14^ Given that UV radiation is an important cause of hyperpigmented lesions, the use of these carriers probably can be beneficial in this case as well. Additionally, NLCs can enhance the penetration of the drugs into the skin by various mechanisms such as direct exposure to the skin surface, skin hydration, lipid exchange between NLCs and SC, and internalization into follicles and adipose tissue.^15^

 Here, for the first time, we aimed to prepare a SEPI-loaded NLC formulation as topical drug delivery system. We investigated the physicochemical properties, long-time stability, efficiency of drug incorporation, drug release pattern, and antityrosinase activity of SEPI-NLC. Moreover, to assess the accumulation and penetration efficiency of SEPI in the epidermis, the cumulative amount of penetrated drug in emulsion, NLC suspensions, and alcoholic solution were studied and compared.

## Materials and Methods

 N-undecylenoyl-phenylalanine (SEPI) was obtained from Seppic, Inc. (Fairfield, NJ, USA). Glyceryl palmitostearate (Precirol® ATO 5) (GPS), glyceryl monostearate (GMS), and cetyl palmitate (CP) were kindly gifted from Gattefosse (France). Poloxamer 188 (POL188), beeswax (BW) and Tween 80 were obtained from Uniqema (Belgium). Medium-chain triglyceride (MCT oil) and Span 80 (SP80) were purchased from Sigma-Aldrich (Germany). Resazurin (Alamar blue), dimethyl sulfoxide (DMSO), L-tyrosine, Mushroom tyrosinase, L-3,4-dihydroxyphenylalanine (L-DOPA), and RPMI1640 cell culture medium were taken from Sigma-Aldrich (St Louis, MO, USA). Fetal bovine serum (FBS), trypsin, and penicillin-streptomycin were purchased from Hyclone (Logan, UT, USA). All chemicals used were of the highest analytical grade and purity. The cell lines of human dermal fibroblasts (HDF) and murine melanoma (B16F10) were taken from the Pasteur Institute of Iran.

 Male BALB/c mice were also obtained from Animal Resources Center at the Pasteur Institute of Iran (Tehran, Iran) and were treated in accordance with the National Institutes of Health guide for the care and use of laboratory animals (NIH Publications No. 8023, revised 1978). The Institutional Animal Care and Use Committee of Mashhad University of Medical Sciences (IR.MUMS.SP.1396.184) approved the animal studies.

###  Preparation and characterization of SEPI-loaded NLCs, SEPI-EMOLSION, and SEPI-ETHANOL

####  Preparation of NLCs

 Different concentrations of GPS, GMS, BW, and CP as solid lipid and POL188, SP80, and Gelucire as the surfactant, alone or in combination, were used to produce the different NLC formulations (Table 1). In all formulations, MCT oil was the liquid lipid of choice. High-speed homogenization and ultrasound methods were used to prepare SEPI-NLCs. In brief, the lipid phase consisting of solid and liquid lipids, SEPI, and surfactant, and the aqueous phase containing water with/without surfactant were separately prepared in a hot water bath (80°C). The aqueous phase was quickly supplemented to the lipid phase and a homogenized emulsion was obtained by an Ultra-Turrax T25 (IKA T10, Germany) at 11 500 RPM for 3.5 minutes. A probe sonicator (Bransonic, USA) was used to disperse the solution for 5 minutes.^16^ The resultant NLCs were chilled to room temperature. The optimal formulation was selected regarding the particles size, polydispersity index (PDI), zeta potential, drug leakage, and homogeneity during one month (Table 2).

**Table 1:** Different preparations of drug free NLC and SEPI-loaded NLC

**Formulation**	**Lipid (%w/v)**				**Surfactant (%w/v)**				**Particle size (nm)**	**PDI**	**Z-potential** **(mv)**
	**GPS**		**GMS**	**BW**	**CP**	**POL188**	**SP80**	**Glucire**			
Non SEPI	1	3.5	-	-	-	2.5	-	-	138.5 ± 2.34	0.166 ± 0.030	-26.8 ± 4.35
	2	3.5	-	-	-	5	-	-	111.8 ± 3.21	0.227 ± 0.08	-27.8 ± 3.21
	3	-	1.75	1.75	-	-	0.75	1.75	140.0 ± 2.98	0.179 ± 0.05	-21.1 ± 4.67
	4	-	1.75	1.75	-	-	1.5	3.5	85.09 ± 3.31	0.262 ± 0.07	-20.7 ± 5.31
	5	-	-	-	3.5	-	0.75	1.75	186.4 ± 4.04	0.170 ± 0.06	-20.9 ± 3.56
0.4 % w/v SEPI	6	3.5	-	-	-	2.5	-	-	172.0 ± 4.43	0.235 ± 0.01	-19 ± 1.5
	7	3.5	-	-	-	5	-	-	180.1 ± 5.01	0.232 ± 0.03	-20.6 ± 2.86
	8	-	1.75	1.75	-	-	0.75	1.75	128.2 ± 3.99	0.189 ± 0.02	-22.2 ± 1.6
	9	-	1.75	1.75	-	-	1.5	3.5	68.41 ± 3.34	0.287 ± 0.02	-22.1 ± 2.1
	10	-	-	-	3.5	-	0.75	1.75	214.1 ± 2.45	0.363 ± 0.03	-39.3 ± 1.7

* In all formulation, MCT oil 1.5% was used as liquid lipid and the volume of water was 10 mL.

**Table 2:** Physiochemical stability of SEPI-loaded NLC formulations after one month

**Formulation**	**Particle size (nm)**	**PDI**	**Z-potential**	**Drug precipitation**	**Phase separation**
SEPI-NLC1	170.8 ± 3.21	0.228 ± 0.02	-18.3 ± 1.78	Yes	Yes
SEPI-NLC2	180.0 ± 2.14	0.242 ± 0.08	-19.6 ± 3.28	No	Yes
SEPI-NLC3	130.1 ± 2.82	0.270 ± 0.04	-21.1 ± 1.54	Yes	No
SEPI-NLC4	62.12 ± 3.53	0.256 ± 0.01	-20.8 ± 2.01	Yes	No
SEPI-NLC5	281.1 ± 4.11	0.320 ± 0.07	-34.3 ± 1.87	No	Yes

 SEPI-EMULSION (SEPI-CREAM), a conventional formulation, was produced based on the equivalent concentration of SEPI, MCT, solid lipid, and surfactant at NLC formulations by hand mixer and without Ultra-Turrax homogenization and probe sonication. MCT, GPS, and SEPI as lipid phase, and dissolved POL188 in water as aqueous phase were quickly mixed. To prepare SEPI-ETHANOL, an equivalent concentration of SEPI in the other formulations was dissolved in 10 mL ethanol.

###  Particle size and PDI 

 Particle size, zeta potentials, and PDI of SEPI-NLCs were measured in triplicate employing Zetasizer Nano-ZS (Malvern Instruments Ltd., UK) by the photon correlation spectroscopy method at 25°C.^17^ Zeta potential reflects the electric charge on the particle surface and physical stability of colloidal systems.This can be determined by the electrophoretic mobility of particles and colloids dispersed in a liquid. Particles with a large negative or positive zeta potential (more negative than −30 mV or more positive than + 30 mV) will repel each other and will not aggregate.^17^

###  Encapsulation efficiency (% EE) and drug loading (%DL) 

 The percentage of the encapsulated SEPI within NPS (% EE) was identified by the indirect method. Briefly, 1.5 mL of SEPI-NLCs suspension was added to an Amicon ultrafilter with a molecular weight cut-off 100 kDa and centrifuged for 30 minutes at 13 000 RPM, by refrigerated centrifuge (Remi Elektrotechnik Ltd., Maharashtra, India) to separate the aqueous and lipid phases. To determine the percent of EE, the sample that was passed through the filter membrane was quantified by high-performance liquid chromatography (HPLC).^16^ HPLC was set on a Lichrospher C8 column (5 Am, 4.6 mm ID25 cm, Hanbang, Dalian, China). The mobile phase was a mixture of methanol: water (95:5 v/v) with 2 mL/min flow rate, 211 nm as the detection wavelength, and the retention time ~1 minute. The calibration curve of SEPI was showed a linear response (R^2^ = 0.9999) in the SEPI concentrations, which ranged from 0.975 to 125 μg/mL. Based on the procedure, the whole value of SEPI in NLCs and weight of NLCs were estimated and drug loading as well as the encapsulation efficiency (%) were calculated based on the following equations:


$$\begin{array}{l} \%\;EE=\frac{Amount\;of\;drug\;added-Amount\;of\;free\;drug}{Amount\;of\;drug\;added}\times 100\\ \%\;DL=\frac{Amount\;of\;drug\;added-Amount\;of\;free\;drug}{Amount\;of\;lipid\;added}\times 100 \end{array}$$

###  Differential scanning calorimetry (DSC) assay

 DSC study was performed using a Mettler DSC 821e (Mettler-Toledo, Gießen, Germany). The scanning was performed at a heating rate of 5°C/min in 0°C–200°C under 5 mL/min nitrogen flow rate and compared with an empty aluminum pan as reference.^18^ Samples involved in bulk PRES, SEPI alone, lyophilized drug-free NLCs, and lyophilized SEPI-NLCs. The thermograms of samples and the calorimetric parameters were calculated by STARe software.

###  Transmission electron microscopy (TEM) imaging

 The morphology of the SEPI-NLCs was investigated by TEM (Zeiss, Jena, Germany). 20 μL drop of diluted SEPI-NLC suspension (1:25) was fixed on a copper grid and dried at 25°C, and then was stained with 2% (w/v) uranyl acetate.^19^ The dried specimen was evaluated by TEM.

###  Long-term stability studies

 The NLCs samples were stored in sealed tubes and away from light at 25°C. The mean diameter of particles, PDI, zeta potential, and clarity of the samples were examined in triplicate 1, 3, 6, and 9 months after preparation. The stability of the SEPI-loaded NLC was also assessed by samples centrifugation at 13 000 RPM for 30 min.^20^

###  In vitro release study 

*In vitro* release study of SEPI from NLC and emulsion formulations was measured by the dialysis bag-diffusion way using a dialysis membrane with a 12-14 kDa molecular weight cut-off (Visking® dialysis tubing, Servia, Greece). Dialysis bags were waterlogged overnight and packed with 1 mL of 0.4% SEPI-NLCs and 0.4% SEPI-EMULTIONs suspensions. The sealed dialysis bags were located into the release buffer (40 mL phosphate-buffered saline (PBS), pH 7.4 with 1% w/w Tween 80) and incubated at 37 ± 0.5 °C, with a stirring speed of 200 RPM. The release test was performed at 0.5, 1, 2, 4, 6, 8, 12, 24, 48, and 72 hours. In any sampling time, withdrawal buffer was exchanged with fresh buffer.^21^ The released SEPI from formulations was analyzed by the HPLC technique as previously mentioned. The mathematical modeling of release kinetics is interpreted with zero-order, Higuchi, and Korsmeyer-Peppas (Ritger–Peppas) as mentioned respective to equations 1, 2 and 3.^22,23^


(1) $$K_{o}t+Q_{o}=Q_{t}$$

(2) $$K_{H}.\;t^{0.5}=Qt$$

Q_t _and Q_o_ symbolize drug concentrations at times t and zero, respectively. K_o_ and K_H_ are constants of Zero-order and Higuchi, respectively.


(3) $$K_{p}t^{n}=M_{t}/M_{\infty }$$

In this formula, M_t_ and M_∞ _ denote the released drug at times t and ∞, “K_p_”, and “n” show the pseudo-kinetic constant and the release exponent, respectively. The n < 0.43, 0.43 < n < 0.89, and n > 0.89 indicated a Fickian diffusion, non-Fickian, and zero-order release, respectively.^24,25^

###  Skin penetration and retention study

 The skin permeation and retention tests were performed *ex vivo* for SEPI-NLCs, SEPI-EMULSION suspension (as conventional formulation), and SEPI-ETHANOL solution employing a 4-station Franz diffusion cell (PermeGear, Inc., USA). The shaved and full-thickness grafts of abdominal skin of BALB/c male mice (7 weeks, 25 g) were soaked in PBS through the dermal side for 1 h to remove the subcutaneous fatty tissues. The Franz diffusion cells were kept at 37°C connecting with a circulatory water jacket. The skins were fixed between the receptor and the donor chambers whereas the SC and dermis sides were exposed to outside the Franz diffusion cell, and the receptor site, respectively. The receptor compartment with 4.54 cm^2^ diffusion area was packed with 25 mL PBS (pH 7.4) contained 1% (w/w) Tween 80 which was stirred at 200 RPM. The skin surface in the donor chamber was coated with a 4000 μg equivalent of SEPI-NLCs, SEPI-EMULSION suspension, and SEPI-ETHANOL solution. The cell was covered with paraffin to prevent evaporation. Next, 2 mL of the solution was withdrawn from the receiver compartment as samples at 0.5, 1, 2, 4, 6, 8, 18, and 24 hours, exchanged with the new PBS and 1% Tween and incubated at 37°C. After 24 hours, the residual samples were collected from the skin surface and dissolved in methanol and chloroform in a 1:2 ratio. All samples were passed through an aqueous 0.45 μm pore diameter membrane filter and the supernatant was assayed by HPLC.^16^ The accumulation of SEPI in the skin (retention rate) was equal to the diminishment in the content of SEPI, which was penetrated into the skin (receptor part) from the SEPI remaining on the skin (residue).

###  Cell viability assay

 The resazurin (or Alamar Blue) was used for cell viability assay. B16F10 and HDF cells were seeded into 96-well plates (4 × 10^5^ cells/well) and cultured in RPMI 1640 and DMEM 1640, supplemented with 10% FBS, 100 μg/mL streptomycin, and 100 U/mL penicillin. The media were maintained in a humidified condition containing 5% CO_2_ for 48 hours at 37°C. Next, the cells were treated with 20 µL resazurin (14 mg/dL) along with 1.25, 2.5, and 5 µM of SEPI-solution and further incubated for 48 hours. The cell viability rate was measured in triplicate by tracking the absorbance at 570 and 600 nm compared to doxorubicin (DOX) 5 μg/mL as a positive control and non-treated cells as a negative control.^26^

###  The assessment of mushroom tyrosinase activity 

 In a 96-well plate, 20 µL of SEPI at concentrations 5, 2.5, 1.25 µM were mixed with 160 µL of L-DOPA (5 mM, pH 6.8). Then, 20 µL mushroom tyrosinase was added to the wells and shaked for 5 minutes. Kojic acid and cell-containing media were used as the positive and negative control, respectively. The plates were incubated at 37°C for 30 minutes. Then, the absorbance of produced dopachrome was checked at 490 nm by an ELISA reader.^26^

###  Cellular tyrosinase activity assay 

 B16F10 cells tyrosinase activity was monitored by determining the L-DOPA oxidation rate to dopachrome. The 24 hours incubated B16F10 cells were seeded in a 12-well plate (10^5^ cells/well) and treated with 5, 2.5, 1.25 µM of SEPI for 24 hours. After that, the cells were resuspended with trypsin and the pellet was washed with PBS. Then, 100 µL sodium phosphate buffer 100 mM (pH 6.8), containing 1% Triton X-100 and 0.1 mM PMSF, was used for 30 minutes to lyse the cell pellets. The lysed cells were then centrifuged for 20 minutes at 10 000 RPM at a cool temperature. 100 µL suspension of protein was mixed with the same amount of 5 mM DOPA in each well and incubated at 37°C for 2 hours. The produced dopachrome was quantified based on its absorbance at 475 nm and the standard curve of mushroom tyrosinase.^26^

###  Date analysis

 Statistical analysis was carried out to evaluate differences between groups using one-way analysis of variance (ANOVA) for cellular test and two-way ANOVA for release and permission studies with GraphPad Prism 6.01 (GraphPad Software, Inc., USA). The data are displayed as a mean ± standard deviation (SD). Comparisons of two groups at similar times were performed by Sidak multiple comparison test. *P* values less than 0.05 were considered as statistical significance.

## Results and Discussion

###  Optimization and characterization of NLC

 The size, shape, colloidal stabilization, and drug loading capacity of the nano-sized carriers are affected their performance. Particles with less than 200 nm size are considered to be optimum size distribution, which are enhanced drug accumulation in action site.^11^ Moreover, the highly negative and positive zeta potentials of NPs provide a high stabile and colloidal nanocarrier. The stable colloidal nanocarriers provide proper properties for NPs in drug delivery applications.^14^ The therapeutic agent may be encapsulated into nanovehicles. Encapsulation of drugs in a nanocarrier may be increased the uptake and delivery of them.^17^ The using nanocarriers with high drug loading capacity is crucial for drug efficacy as the suitable drug concentration will release from nanocarriers at the right time in the right place.^17^ The optimized formulation was selected based on these criteria.

 In this study, an optimal formulation with appropriate physicochemical features was obtained among five formulations with different ratios of lipid and surfactant, with and without SEPI. The particle size, PDI, zeta potential, and long-term stability were used for selection of optimized SEPI-loaded and drug-free formulation (Tables 1 and 2). The lipid/surfactant ratio may influence the size, zeta potential, % EE and loading of SEPI into SEPI-NLCs. The optimal nanocarrier defines as a carrier with a small particle size, higher loading content, more cumulative drug release profile, and better physicochemical stability.

 According to Tables 1 and 2, SEPI-NLC2 was selected as the optimal formulation. In comparison with SEPI-NLC2, a remarkable increase in particle size and PDI were detected via adding the SEPI to NLC5. SEPI-NLC3 and SEPI-NLC4 showed drug precipitation, non-homogeneous state during 1 month, instability, and large size distribution ranging from 10 to 400 nm. SEPI-NLC1 demonstrated a few drug precipitation during storage time.

 The long-term stability of optimal formulation of SEPI-NLC in room temperature during 9 months indicated good stability and the slight change in particle size, z-potential, and PDI (Table 3). No notable alteration of clarity and phase separation was observed.

**Table 3:** Report of SEPI-CLNs stability during 9 months

**Time (month)**	**Particle size (nm)**	**PDI**	**Zeta potential (mv)**
0	180.1 ± 5.01	0.232 ± 0.03	-20.6 ± 2.86
1	180.0 ± 2.14	0.242 ± 0.08	-19.6 ± 3.28
3	182.8 ± 2.86	0.240 ± 0.06	-28.7 ± 3.51
6	197.1 ± 3.96	0.256 ± 0.01	-24.1 ± 4.42
9	210.4 ± 4.73	0.269 ± 0.06	-21.6 ± 4.61

 The calculated encapsulation efficiency and drug loading of SEPI-NLCs were 90.81 ± 3.75 and 7.26 ± 3.30, respectively. GPS and GMS are lipid excipients for the construction of sustained-release dosage forms due to their lipophilic criteria. This amorphous diglyceride with two different long-chain fatty acids (C16, C17) forms a disordered lipid lattice. The combination of this glyceride with other glycerides provides extra space for drug loading due to no crystal defects and the crystalline structures.^27^ It explains the high entrapment efficacy of SEPI-NLC. The emulsified nature of GPS can also reduce particle size and increase drug release. In addition, PRES is an anionic lipid with the OH functional group in its structure and prevents aggregation of NPs and instability during storage.^10^ It has been shown that the lipid amount up to 5% in NLC formula increase the particle size in the range of micro-particles.^28^

 The liquid lipids with a shorter carbon chain length, such as MCT oil provide more capacity for drug loading resulting in increasing the release rate. Additionally, according to Einstein-Stokes law, adding liquid lipid to solid lipid in the molten state can increase drug release from NPs due to decrease viscosity.^29^ Moreover, increasing the ratio of liquid to solid lipids prevents particle agglomeration and increasing the size of NPs. We used the 70:30 ratio of solid to liquid lipids.

 Suitable surfactant causes high, uniform, and long-term lipid dispersion in the aqueous phase.^30^ POL188 is a non-ionic surfactant with minimal skin irritation, which is used in the preparation of NPs.^31,32^ POL188 was selected as a hydrophilic surfactant due to its wide melting points from 52 to 57 °C approximately nearby the GPS melting point (56°C). POL188 have a high value of hydrophile lipophile balance (HLB) and reduces the surface tension between the lipid excipient and the resolving medium. Additionally, GPS has a hydrophobic character expressed by the low HLB value of 2 that can lead to slow drug release and POL188 can help to modify the drug release from the lipid matrix structures.^33^ Increasing the surfactant concentration reduces the particle size because of the decrease of surface rigidity between the solid lipid and the external liquid environs.^34^

###  DSC investigations

**Figure 2:**
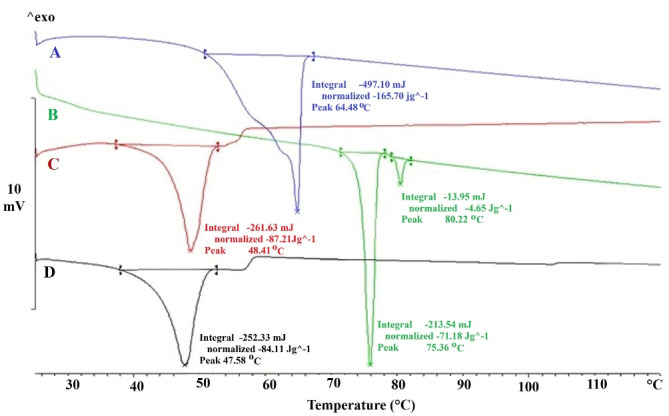
DSC heating curves of Glyceryl palmitostearate (A), Sepiwhite^®^ (B), drug-free NLC (C), and SEPI-NLC (D).

Figure 2 illustrates the DSC thermograms of SEPI, GPS, drug-free NLC, and SEPI-NLC. The thermogram of SEPI-NLC did not indicate the melt-crystallization peak of SEPI around 75°C, revealing the amorphous state of SEPI in NLC.^35^ That also means the drug has completely entered into NPs. Since the drug was freely present in the formulation, it should be localized on the external surface of NPs during the lyophilization process and the endothermic peak of lyophilized SEPI-NLC may display around 75°C. The high percentage of drug encapsulation (90.815 ± 4.631) confirms this.^18,36^ SEPI-NLC showed one peak at 48.41°C that was lower than GPS by melting point of 64.48°C and SEPI that exhibited two peaks at 75.36°C and 80.22°C. This phenomenon is raised from the formation of nanoscale particles instead of the bulk lipid as well as dissolving the drugs, surfactants, or oils in the lipid matrix.^37^

 The lipid NPs are usually dispersed in size with different melting points (smaller particles ↔less melting point).^30^ According to Figure 2, the enthalpy of GPS being much higher than that of lyophilized drug-free NLC (165.70 J/g in lipid bulk versus 87.21 J/g in drug-free NLC) that suggests a loss of crystallinity of the lipids in NLCs formula and crystal dis-ordering that may affect the loading capability.^38^

###  Morphology of SEPI-loaded NLCs

**Figure 3:**
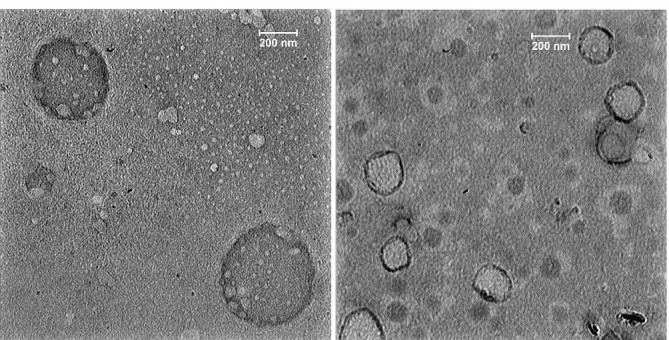
TEM images of optimized formulation of SEPI-NLCs (NLC containing Sepiwhite^®^).

 The TEM images showed that the mean diameter of SEPI-NLC was less than 200 nm, which was confirmed by the DLS-based analysis (Figure 3). The polydispersity can be probable dissimilarity of particle size in the particle size analyzer and TEM assay.^37^ The images of SEPI-NLCs demonstrated a spherical nanosized shape and a small size distribution.

###  In vitro release study

 There is a hypothesis that lipid NPs > 100 nm are not able to penetrate into the SC layer and stay on the surface because of their dimensions and rigidity. In contrast, they may affect penetration through the hair follicles, confirming the role of other mechanisms in drug penetration.^15,39,40^ Furthermore, some NPs may be found intact only in the first layer of the SC.^10^ Therefore, the release of the encapsulated drug is necessary for optimal topical drug delivery. The blocking of α-MSH and β-ADR receptors on the cell surface are known as the main SEPI mechanism. So, when NPs penetrate to the deepest epidermal layer, the drug must be released from the NPs before entry the cells to target the receptors.

**Figure 4:**
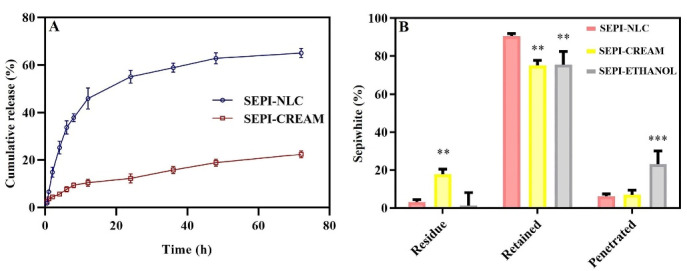
**(A)** In vitro release curve of SEPI-loaded NLC and SEPI-EMULSION; **(B)** Ex vivo release of SEPI-NLC, SEPI-emulsion and SEPI-ethanol after 24 hours. The data is described as mean ± SD of the triplicate test; ** P < 0.001; ***P < 0.001.

 The *in vitro* release pattern study provides an idea for the dose and time of therapy in the *in vivo* stage. As represented in Figure 4A, SEPI-NLCs verified a biphasic release profile that presented a burst release during the first 4 hours, afterward a prolonged release up to 72 hours. About 65% of SEPI was released from SEPI-NLC within 72 hours, while in SEPI-EMULSION, this value was 23%. So, a faster release rate of SEPI from NLC was observed compared to SEPI-EMULSION. Olejnik et al. investigated the *in vitro* release of SEPI from topical formulations consisting two different macroemulsions, carbomer- and hydroxyethylcellulose-based hydrogels, and microemulsions.^8^ A comparative analysis showed the highest release rate of active substance from the carbomer hydrogel. After 10 hours, about 80% of the SEPI was permeated through the membrane. However, a continuous release of active compound was observed for hydroxyethylcellulose hydrogel, which after 24 h reached about 80%. Additionally, it can be noted that much more SEPI was released from macroemulsion #2 (prepared by Oleic acid) than macroemulsion #1 (prepared by Isopropyl myristate). After 24 hours, 60% of total mass of active compound permeated to the receptor medium, while in the case of macroemulsion #1 only 20% of SEPI was released. Regarding these, it can be concluded that hydrogels can be better vehicle for the SEPI than macroemulsions.^8^

 Burst release occurs when the solubility of the drug in the molten lipid is lower than its saturation limit, so the drug was enhanced in the outer shell of the particles and formed a drug-enriched shell model.^41^ Poloxamer is a hydrophilic surfactant that acts as a pore-forming agent causing the penetration of the dissolution medium. The penetrated medium can dissolve the localized drug in the outer shell resulting in burst release at the beginning of the test.^33^ Moreover, increasing the temperature of the NLC formula during the test up to 37°C initiate β modification as a trigger and expulsion of the drug to the water phase of the formulation due to more ordered lipid particle matrices (trigger-controlled release) and increase the diffusion coefficient (according to Einstein-Stokes law) of the drug in the early hours.^29^ The solid lipid matrix plays a role in prolonging release.^37^

 According to Figure 4A, SEPI-NLC shows further and more complete cumulative drug release than SEPI-EMULSION because of the smaller size of NPs than emulsion (micro-sized). In other words, small size in NPs improves the surface-area-to-volume ratio and decreases the release pathway in NPs.^30^ Indeed, the burst release in micro-particles reduces, and the release is prolonged and incomplete. The drug release from the matrix of systems is controlled by diffusion or digestion, single or mixture, manners. Further investigation showed the drug release from the lipid matrix follows the Korsmeyer-Peppas model (n = 0.358 and n < 0.43) and Fick’s first law. According to this law, diffusion occurs in response to a concentration gradient (diffusion model). The process of drug release with linear regression was also studied. Biphasic release pattern is the optimal pattern for topical drug delivery because the initial fast release can simplify SC permeation of SEPI by providing an appropriate concentration, while the sustained release keeps up the local concentration; induce a long anti-melanogenesis effect of SEPI.

**Figure 5:**
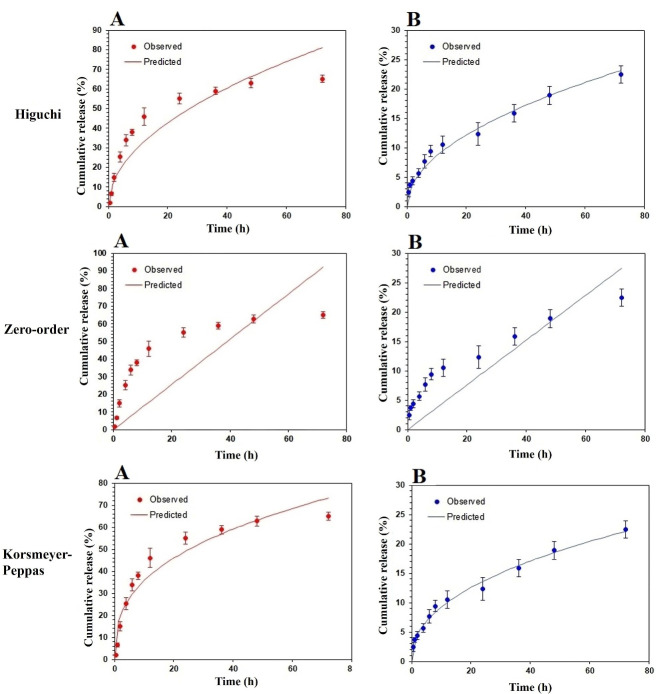
Simulation of % cumulative *in vitro* release of SEPI from SEPI-NLCs **(A)** and SEPI-EMULSION **(B)** vs. time using zero-order, Higuchi, and Korsmeyer-Peppas.

 The kinetics models including Korsmeyer-Peppas, Higuchi, and zero-order employing to analyze the cumulative *in vitro* drug release have shown in Figure 5. Based on the Korsmeyer-Peppas model, the “n” rate was obtained 0.364 and 0.441 at SEPI-SLN and SEPI-EMULSION samples, respectively, indicating the Fickian diffusion model. In addition, the higher regression coefficient (R^2^) was observed at the model of Higuchi for SEPI-SLN and SEPI-EMULSION as compared to the zero-order model, revealing the diffusion as the dominant release mechanism (Table 4). The K values (K_o_, K_H_, and K_p_) of SEPI-SLN were higher than SEPI-CREAM, which is signified the fast release of SEPI from NLC.

**Table 4:** The parameters of SEPI release kinetic models.

**Model**	**SEPI-NLC**			**SEPI-CREAM**		
	**R**^2^	**n**	**K**	**R**^2^	**n**	**K**
Zero-order	0.1257	1.0	1.282	0.5248	1.0	0.381
Higuchi	0.8281	0.5	9.550	0.9456	0.5	2.721
Korsmeyer-Peppas	0.9056	0.364	15.491	0.9701	0.441	3.383

###  Ex-vivo skin permeation study

 To evaluate *ex vivo* skin permeation of drug-loaded in SEPI-NLC was compared with SEPI-EMULSION and SEPI-ETHANOL formulations by Franz diffusion cells method. According to Figure 4B, the rate of residual drug in SEPI-ETHANOL was lower than two other formulations, while the penetrated drug was high. Alcohols may damage the SC by extracting and dissolving keratin and other compounds in this layer, thereby increasing the absorption of drugs.^40,41^ In addition, the rapid permeation or evaporation of ethanol during the test can enhance the concentration of SEPI in solution with increase thermodynamic activity of SEPI.^42^ Generally, the system tends to reduce increased thermodynamically activity by promoting the diffusion of the drug into the skin resulting in the further penetration of the drug.^42^ These factors lead to the higher permeation of SEPI through the skin and less SEPI remaining on the skin from SEPI-ETHANOL. The higher percentage (23.2%) of the drug enters into the buffer medium (equivalent to systemic blood flow) from SEPI-ETHANOL compared to the emulsion and NLC (6%-7%) formulations. Therefore, SEPI-ETHANOL moves away from its target area (basal epidermis) and can maximize the risk of systemic uptake and adverse effect in topical use.

 SEPI-loaded NLCs localized and accumulated a high amount of drug in the skin with a low residual drug on the skin surface and penetrated through the skin at the end of the permeation study. NLCs can promote drugs skin permeation more than EMULSION form by various mechanisms such as lipid exchange between NLC and SC lipids, and stronger SC occlusive effect by forming a continuous thin film on the skin due to its smaller particle size.^15^ The occlusion effect prevents the evaporation of skin water resulting in the hydration of the SC. A higher drug release rate along with more gradient concentration could also significantly increase the accumulative and permeation of SEPI into the skin in NLC (up to 88.8%) more than EMULSION (65%) and ETHANOL formulations (74.8%) (*P* < 0.01), whereas no significant difference was observed in SEPI accumulative level in the skin between SEPI-EMULSION and SEPI-ETHANOL. The residual amount of SEPI on the skin after 24 hours in the NLCs formulation (4.9%) was much less than the SEPI-EMULSION (27.9%) (*P* < 0.01).

 In SEPI-NLC aqueous dispersion, after 24 h, only 55.8% SEPI was released, while 88.8% SEPI was retained into the skin in the *ex vivo* penetration study (Figure 4B). The increased epidermal permeability of SEPI may be related to the close association of skin lipids with NLC surfactants and lipids.^20,29^ The poor water solubility of SEPI can avoid releasing the SEPI into the buffer medium, while the release of the drug on the skin surface can promote by enzymatically lipid degradation and electrolyte change in the SC. Indeed, electrolyte change together with increasing temperature up to 37°C could cause higher-ordered assembly in the lipid particle by low-energy β modifications and drug expulsion.^42^ Ultimately, all of these issues assist to localize the SEPI-NLC in skin layers. Generally, release and permeation studies show that NLCs enhance the drug permeation.

###  SEPI cytotoxic effect 

**Figure 6:**
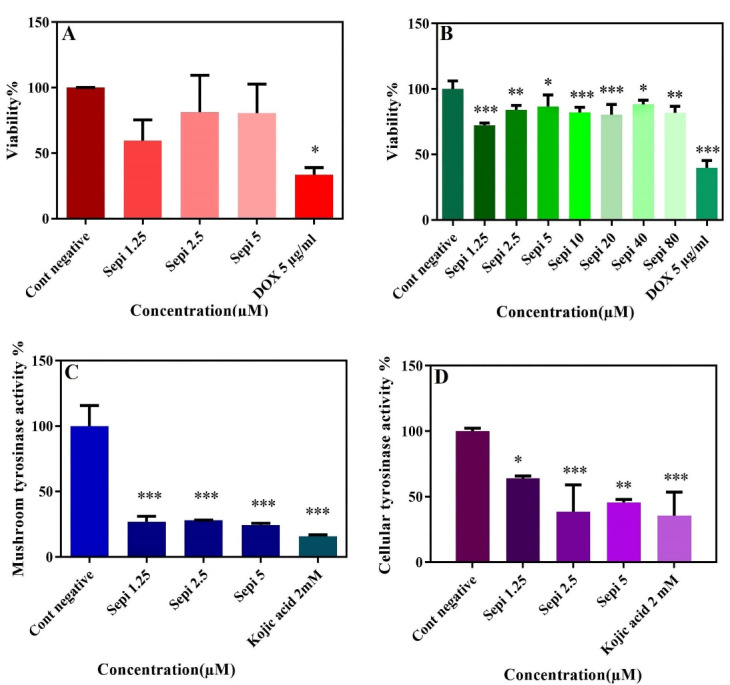
Effect of the SEPI concentration on cell viability of B16F10 **(A)** and HDF **(B)** cells in comparison with DOX as positive control; Effect of the SEPI concentrations and kojic acid as positive control on mushroom tyrosinase activity **(C)** and cellular tyrosinase activity **(D)**. Values are presented as mean ± SD for triplicate. *** P < 0.001, ** P < 0.01, and * P < 0.05.

 The cytotoxicity of SEPI concentrations on cell viability of B16F10 and HDF cells showed no significant difference between all groups with negative control (*P* < 0.05). DOX was used as a positive control due to a great inhibitory effect on cell proliferation.^26^ The result confirmed that non-cytotoxic effect of SEPI on B16F10 and HDF cells at the tested concentrations (Figure 6A, B). Over 80% of the studied cells survived in the presence of high concentrations of SEPI solution. The use of skin cells in *in vitro* evaluation is safe and cost-effective, and in certain cases, such as examining the effects of toxicity and cellular stimulation, eliminates the need for human or rat skin.

###  Cellular and mushroom tyrosinase activity of SEPI

 Tyrosinase is known as a vital and rate-limiting enzyme in catalyzing melanin biosynthesis.^43^ As shown in Figure 6C, the inhibitory effect of the SEPI concentrations on mushroom tyrosinase activity revealed that 72% of mushroom tyrosinase activity was significantly reduced (*P* < 0.001) in cells treated with SEPI 2.5 µmol/L compared to the untreated control group. This rate was 84% for kojic acid 2 mmol/L as a positive control (Figure 6C). These results displayed the direct inhibitory effects of SEPI on mushroom tyrosinase activity. Given that phenylalanine is the precursor of L-tyrosine as well as similarity of SEPI structure to Undecylenoyl-phenylalanine, SEPI may play a mimic role for the tyrosinase. In addition, we evaluated the anti-melanogenesis effect of different SEPI concentrations on cellular tyrosinase activity to found an intracellular pathway for melanin synthesis inhibition. As shown in Figure 6D, the significant inhibition of tyrosinase activity was observed in the treated cells with 1.25, 2.5, and 5 µmol/L of SEPI solution. These results confirm that the SEPI can reduce melanogenesis through intracellular mechanisms such as tyrosinase inhibition or antagonized pathway of α-MSH and β-AD receptors lead to reducing hyperpigmented lesions.

## Conclusion

 Herein, a nanosized SEPI-loaded NLC was provided with good long-term physical and chemical stability (9 months at room temperature). The DSC analysis also exhibited an amorphous state of SEPI in NLCs. The results indicated an entrapment efficiency of 90.81 ± 3.75%. The release study demonstrated that SEPI-NLC had a biphasic release outline with an initial burst release, since about 65% of SEPI was released from SEPI-NLC within 72 hours. The *ex vivo* permeation profiles revealed that the higher SEPI accumulation in the skin following application of SEPI-NLC (up to 88.8%) compared to SEPI-EMULSION (65%) and SEPI-ETHANOL (74.8%) formulations (*P* < 0.01). In addition, SEPI showed an inhibition rate of 65% for mushroom tyrosinase activity and could inhibit melanogenesis through direct inhibition of tyrosinase activity up to 72% in B16F10 cells. Moreover, results of *in vitro* cytotoxicity assay confirmed SEPI-NLCs to be non-toxic and safe for topical use. NLC was also proved a highly desired nanocarrier for epidermis drug delivery of SEPI. Skin permeation and retention profiles represented that the NLCs formulation allowed efficient SEPI delivery that may be beneficial for sustained antityrosinase activity and a high accumulation of SEPI within the epidermis. These findings suggest that NLCs as an appropriate nanocarrier for brightener delivery to the epidermis.

## Acknowledgments

 The results presented in this paper providing by a Pharm.D thesis (Grant number: 960162) supported by the Nanotechnology Research Center, Mashhad University of Medical Sciences (MUMS).

## Competing Interests

 Authors declare no conflict of interest in this study.

## Ethical Approval

 All of the experiments were ethically approved by the Institutional Ethical Committee and Research Advisory Committee of Mashhad University of Medical Sciences and carried out according to their recommendations under registration number (IR.MUMS.SP.1396.184).
